# Use of Internet/Application-Based E-consults by General Practitioners in Japan to Resolve Patients’ Problems: A Descriptive Observational Study

**DOI:** 10.7759/cureus.76690

**Published:** 2024-12-31

**Authors:** Taku Harada, Toru Morikawa, Hiroki Furuya, Tomoki Sato, Minami Kakehi, Hiroki Yamada, Taro Shimizu

**Affiliations:** 1 Department of General Medicine, Nerima Hikarigaoka Hospital, Nerima, JPN; 2 Department of Diagnostic and Generalist Medicine, Dokkyo Medical University Hospital, Mibu, JPN; 3 Department of General Medicine, Nara City Hospital, Nara, JPN; 4 Department of Clinical Epidemiology, Hyogo Medical University, Nishinomiya, JPN; 5 Research and Development, TANAAKK, Inc., New York, USA; 6 Department of Pediatrics, Hiroshima City Funairi Citizens Hospital, Hiroshima, JPN; 7 Center of Postgraduate Clinical Training, General Hospital Minamiseikyo Hospital, Nagoya, JPN; 8 Research, Medii, Inc., Shinjuku, JPN

**Keywords:** application, digital health technology, e-consult, general practitioner, internal medicine, internet

## Abstract

Introduction

Electronic consultations (e-consults) refer to digital health technologies that enhance clinical information sharing and improve patients’ access to expert healthcare providers. This study aims to describe the current utilization of the Internet/application-based e-consult system and to assess how effectively it resolves clinical questions.

Methods

Participants were general practitioners (GPs) who had installed the e-consult platform on their personal computers or cell phones. Hospital specialists who responded to the e-consults were previously registered on our e-consult platform and also participated in the study. We have developed and implemented an Internet/application-based e-consult system that GPs can use on their personal computers in Japan. In this descriptive study, GPs used the e-consult platform via an Internet/application to consult hospital specialists. The study period was from May 1, 2020, to October 31, 2021. The outcome measure was the resolution rate, calculated by dividing the number of cases resolved solely through e-consults by the total number of e-consult cases. Another outcome measure was the number of cases in which a plan was determined using e-consults. Other outcome measures were GP satisfaction, assessed using a 5-point Likert scale for e-consults, the number of chats required for a hospital specialist to respond, and the time from the GP's initial question to the hospital specialist's first response.

Results

Of the 329 e-consult cases identified, 91 conducted by GPs were included in the final analysis. The number of cases resolved using only e-consults was 47, resulting in a resolution rate of 52% in the study. The minor specialists exhibited the highest resolution rate using e-consults alone. The number of cases where a plan was determined using e-consults was 83, accounting for 91% of the total 91 cases. The level of GP satisfaction with the e-consult process was high, with a median satisfaction score of 5 on a 5-point Likert scale. Most e-consults received their initial response within two hours. The number of chats required for hospital specialists to respond was very low, with a median of only one chat being necessary.

Conclusion

The Internet/application-based e-consult system enabled GPs to address a wide range of medical problems. Further studies with large samples are necessary, even though these results serve as an important benchmark for future research on e-consults in Japan.

## Introduction

Electronic consultations (e-consults) are components of provider-to-provider digital health technology in telemedicine, which enhance clinical information-sharing and improve patients’ access to expert healthcare providers [1,2]. Previous studies reported the reliability, convenience, and efficacy of e-consults; they minimize unnecessary visits to specialists, shorten waiting times, and reduce medical expenditure [3,4]. In addition, the management of common issues has improved over time as users continue to employ e-consults [5,6]. Most users are highly satisfied with e-consults [7], which has also been reported by studies investigating patients’ acceptance of e-consults. Their use has the potential to reduce the burden loaded on primary care services [8]. In addition to facilitating access to care for patients in remote areas and regions with an uneven distribution of hospital specialists, e-consults also help address issues related to the shortage of specialists [9-11]. They were also effective in the setting of the recent COVID-19 pandemic [12,13].

E-consults may benefit general practitioners (GPs) as they are applicable across a wide range of clinical fields [14,15]. In addition, e-consults are predominantly used by GPs and serve as a valuable tool to enhance communication between GPs and hospital specialists [15]. However, their implementation has faced several barriers, including differences in licensing and the provision of infrastructure and resources [16]. Thus, a system that simplifies the implementation of e-consults for GPs is necessary. In addition, traditional e-consult systems have made it difficult for users and specialists to communicate interactively via chat over the Internet. To the best of our knowledge, no previous studies examined the Internet/application-based e-consult system. Moreover, no studies have yet reported on the implementation of e-consults in Japan.

This study aims to describe the current utilization of the Internet/application-based e-consult system and to assess how effectively it resolves clinical questions. In addition, this study aims to describe the actual use of e-consults in Japan.

## Materials and methods

Study design and participants

We conducted a descriptive observational study in Japan. Participants were GPs who had installed the e-consult platform on their personal computers or cell phones. Hospital specialists who responded to the e-consults were previously registered on our e-consult platform and also participated in the study. The study period was between May 1, 2020, and October 31, 2021, and we included cases involving consultations initiated by GPs contacting hospital specialists. We excluded e-consult cases where the e-consult user was not a GP. The definition of GPs includes physicians who deliver comprehensive medical care across a wide range of conditions. This definition does not mandate certification as a family medicine specialist. In Japan, most GPs practice in clinics without holding certification in family medicine. GPs remain anonymous, while hospital specialists are identified by name. The platform includes over 1,500 hospital specialists who guarantee the quality of consultations by sharing their specialties, areas of expertise, and certifications from recognized professional societies. In Japan, most specialists practice as employed physicians in hospitals, and therefore, we consider them to be hospital specialists.

Implementation of e-consults

Medii Corporation has developed a unique chat-based e-consult platform that works on physicians' personal computers/cell phones in Japan [17]. The e-consult platform utilizes a standardized electronic form whereby users submit patient-specific clinical information (excluding personal details) to a professional service. The platform can be operated using either an Internet browser or application, making it usable in various areas throughout Japan. GPs and hospital specialists do not need any special training to use our e-consult system because it is a simple chat-based communication tool that requires no special operation.

The e-consults facilitate chat-based communication between GPs and hospital specialists. Other patient information, such as blood test results and clinical images, is attached as needed. Accordingly, an appropriate hospital specialist is then assigned for a particular e-consult. The hospitalist who first agreed to accept the consultation was assigned to the consultant. The hospital specialist logs into the system to respond to the inquiry via a “consultation note,” and the GP may respond to the hospital specialist’s comment as necessary. In this way, online consultation can be performed. GPs can access the e-consult system at no cost, while hospital specialists are rewarded with points for their responses, which can be redeemed for online shopping. The e-consult system is intended for clinicians to improve the quality of their clinical practice on an individual level, operating separately from insurance-based medical services.

We have shown the conceptual visual design and the actual interface of our Internet/application-based e-consult system as appendix figures (Figures 4-5).

Data, factors, and outcomes

We extracted factors related to e-consult cases, GPs, and hospital specialists during the study period. Factors related to e-consult cases included age, sex, blood tests, clinical imaging, and clinical photography. Factors related to GPs included the number of post-medical school years and e-consults per GP. The factors related to hospital specialists included the number of post-medical school years and the division of the hospital specialist. The index day for the e-consult was defined as the day on which the consultation was initiated by a GP contacting a hospital specialist. We classified the divisions of hospital specialists into internal medicine subspecialties, general internal medicine, surgery, and minor specialists. Minor specialists were defined as physicians practicing in the following specialties: dermatology, pediatrics, emergency care, urology, ophthalmology, otorhinolaryngology, and palliative care.

We obtained information on age, sex, blood tests, clinical imaging, and clinical photography directly from the e-consult platform’s standardized data fields. These details were entered by GPs as part of their routine documentation of each consultation. Similarly, data regarding GPs and hospital specialists, including their professional backgrounds, were extracted from the information they provided at the time of registration on the platform. No additional prompting or survey was used to gather these data.

The outcome measure was the resolution rate, calculated by dividing the number of cases resolved solely through e-consults by the total number of e-consult cases. For example, when a GP consults a hospital specialist about a treatment plan for rheumatoid arthritis, the case is considered "resolved" if the hospital specialist provides an appropriate plan, the GP has no further questions, and the GP confirms their intention to implement the suggested plan.

Another outcome measure was the number of cases determining a plan using e-consults. The “number of cases determining a plan using e-consults” is a composite outcome that includes both cases fully resolved solely through e-consults and those in which a course of action for future diagnosis or treatment was established via e-consults. For example, if a GP consults a hospital specialist regarding a case of fever of unknown origin and, although the exact cause remains unidentified, the specialist's advice leads to the establishment of a plan for further investigations, such as blood cultures or contrast-enhanced CT scans, the case is categorized as one in which a plan was determined through the e-consult.

These outcomes were independently assessed by TH, TM, and HF, who are all internists and researchers. For outcomes where their evaluations differed, decisions were finalized through joint discussion and consensus.

Other outcome measures were GP satisfaction, assessed using a 5-point Likert scale for e-consults; the number of chats required for a hospital specialist to respond; and the time from the GP's initial question to the hospital specialist's first response.

Statistical analysis

We analyzed categorical variables using counts and proportions and continuous variables using medians and interquartile ranges (IQRs). We described the differences in resolution rates among the hospital specialties. Additionally, we described patients’ problems addressed through e-consults across different hospital specialist divisions. All statistical analyses were performed using JMP version 16.0 (SAS Institute, Cary, NC, USA).

Ethical considerations

GPs and hospital specialists performing e-consults obtained individual consent for data use, with each participant confirming their consent through a dedicated checklist integrated into the e-consult platform. This study was approved by the ethical review board of Showa University Koto Toyosu Hospital (approval number: 21-070-A) and was conducted in accordance with the Declaration of Helsinki. Because this was an observational study, the ethics committee determined that individual patient consent was not required and approved the use of an opt-out method. The e-consult platform adheres to the guidelines for the protection of personal medical information issued by Japan's Ministry of Health, Labour, and Welfare.

## Results

Characteristics of e-consults

**Figure 1:**
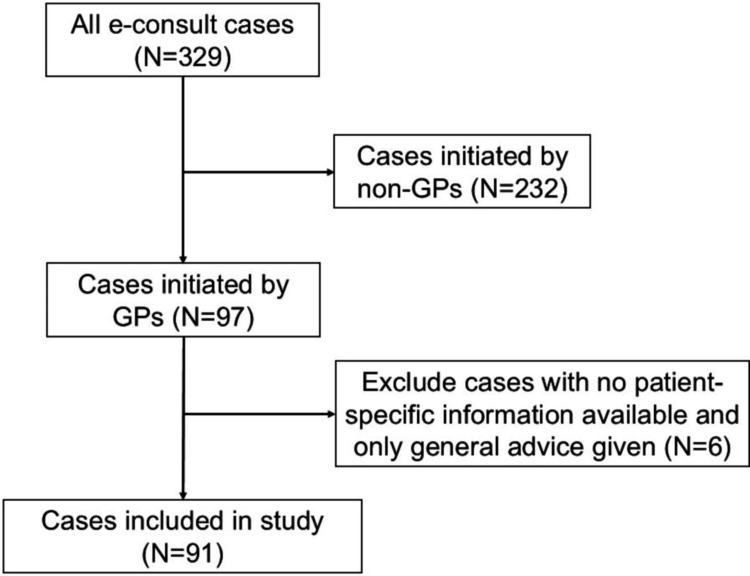
Flow chart of the study

During the study period from May 1, 2020, to October 31, 2021, a total of 329 e-consult cases were identified, of which 97 were performed by GPs. We excluded six e-consult cases where no patient-specific information was available and only general advice was given. Ninety-one cases were included in the final analysis (Figure 1). Among e-consult cases, blood tests were performed in 56 cases (62%), while clinical imaging (X-ray or CT) and clinical photography were performed in 17 (19%) and 12 (13%) of cases, respectively. The median number of post-medical school years of GPs was 8, with a median of 3 e-consults per GP. The majority of hospital specialists were in internal medicine subspecialties: 58 (64%), followed by minor specialists: 18 (20%), general internal medicine: 8 (9%), and surgery: 7 (7%) (Table 1).

**Table 1:** Characteristics of e-consults IQR: interquartile range. *For patients aged 10 or older, only the decade of age is given because more precise information was unknown. ^$^One e-consult involved multiple clinical examinations.

Factors	E-consult cases in the study (N=91)
E-consult-related factors	
Age, median (IQR)*	70 (40-80)
Male, n (%)	42 (46)
Conducted blood tests, n (%)	56 (62)
Conducted clinical imaging (X-ray or computed tomography), n (%)	17 (19)
Conducted clinical photography, n (%)	12 (13)
General practitioner-related factors	
Post-medical school years, median (IQR)	8 (4-26)
Number of consultations per general practitioner, median (IQR)	3 (1-7)
Hospital specialist-related factors	
Post-medical school years, median (IQR)	10 (8-16)
Division of hospital specialist, n (%)	
Internal medicine subspecialties	58 (64)
Minor specialists	18 (20)
General internal medicine	8 (9)
Surgery	7 (7)

Outcomes of e-consults

**Figure 2:**
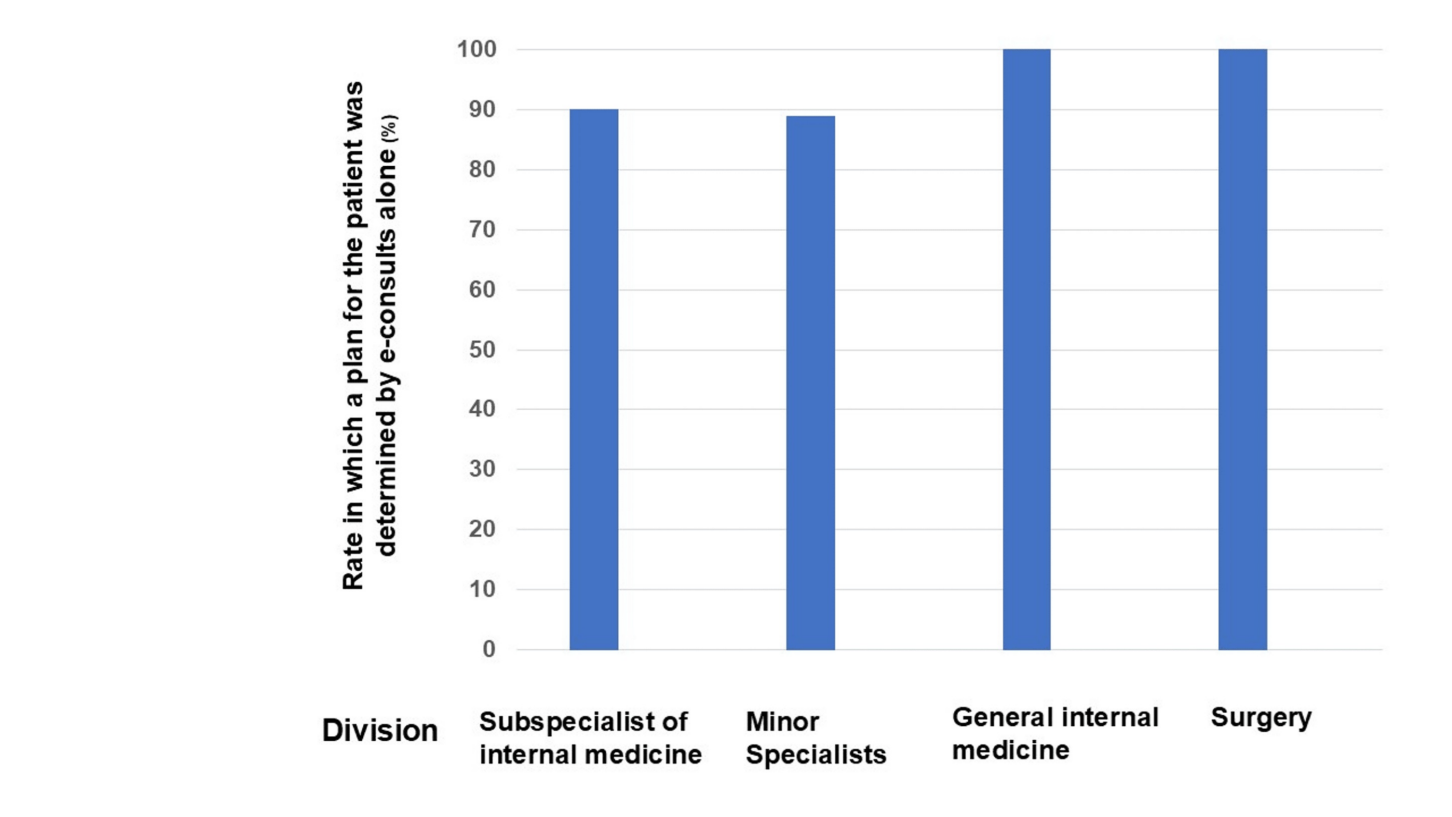
Rate in which a plan for the patient was determined by e-consults alone

The number of cases resolved using only e-consults was 47, resulting in a resolution rate of 52% in the study. The number of cases resolved solely through e-consults, along with their respective resolution rates for each hospital specialist, were as follows: 25 cases (43%) by internal medicine subspecialists, 16 cases (89%) by minor specialists, 2 cases (25%) by general internal medicine specialists, and 4 cases (57%) by surgeons. The minor specialists exhibited the highest resolution rate using e-consults alone. Most problems were resolved solely by e-consults involving minor specialists concerned with rashes diagnosed through clinical photography. Eight e-consults involved GPs contacting dermatologists, and all eight cases were resolved based on clinical pathology using the e-consult platform (Table 2 and Figure 2).

**Table 2:** Outcomes associated with e-consults IQR: interquartile range.

Outcomes	E-consult cases in the study (N=91)
Number of cases resolved using only e-consults, n (%)	47 (52)
Number of cases determining plan using e-consults, n (%)	83 (91)
GP satisfaction with e-consults measured on 5-point Likert scale, median (IQR)	5 (5-5) (n = 75)
Number of chats required for hospitalists to respond to and complete consultation, median (IQR)	1 (1-2)
Minute from the GP's question to the hospital specialist's initial response, median (IQR)	54 (27-145) (n = 90)

**Figure 3:**
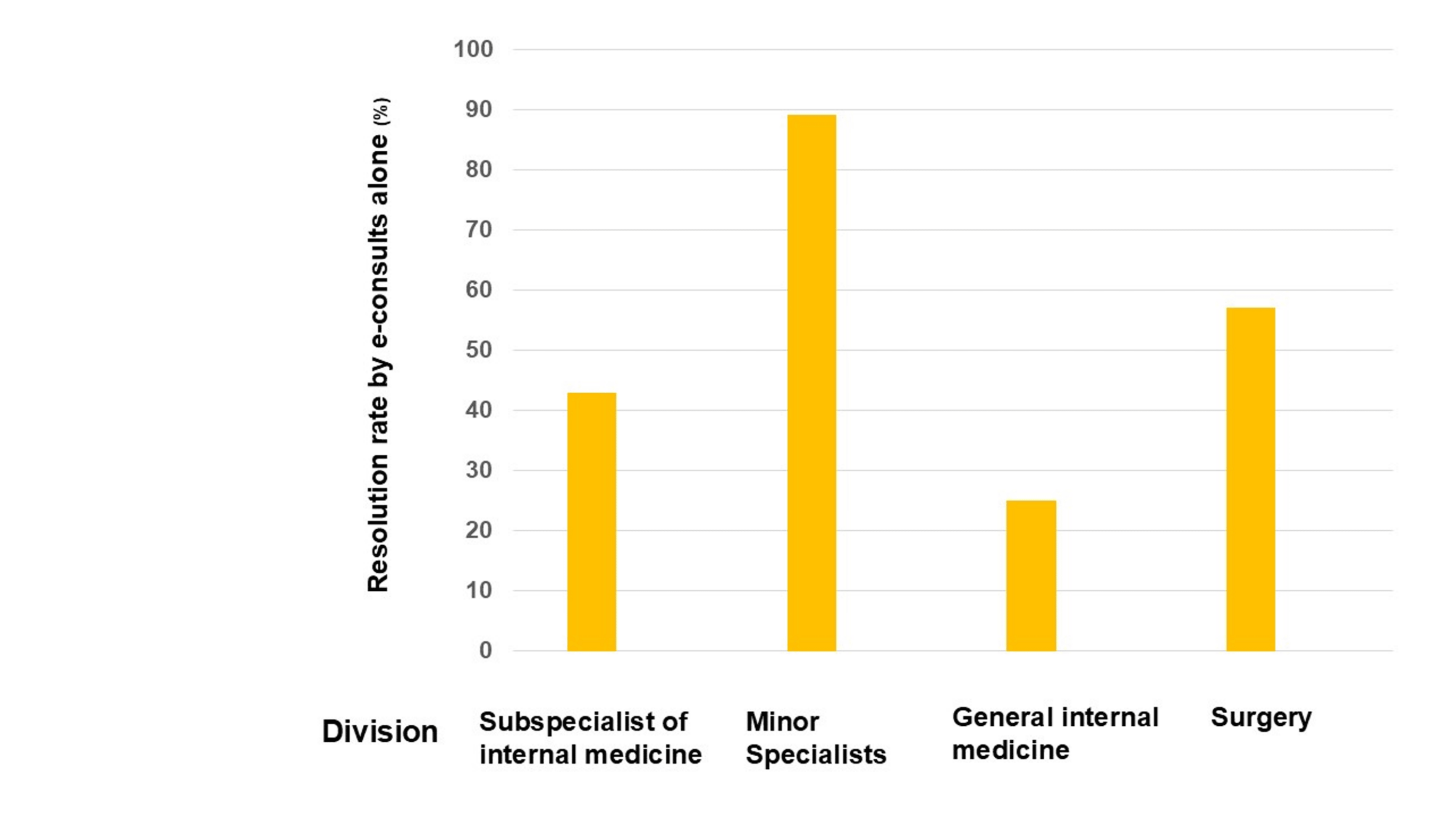
Resolution rate by e-consults alone

**Figure 4:**
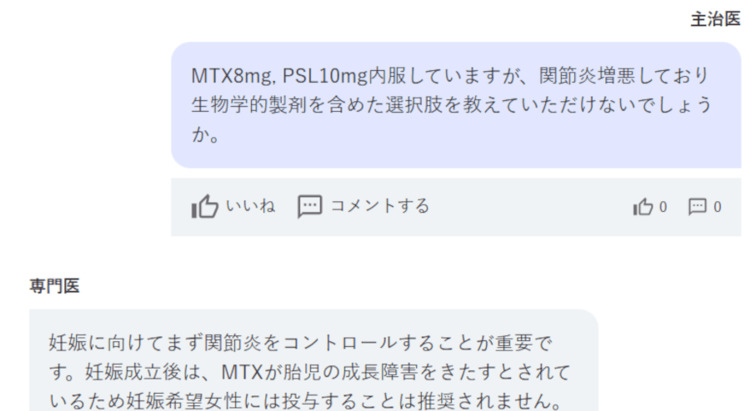
The actual interface of our internet/application-based e-consult system

**Figure 5:**
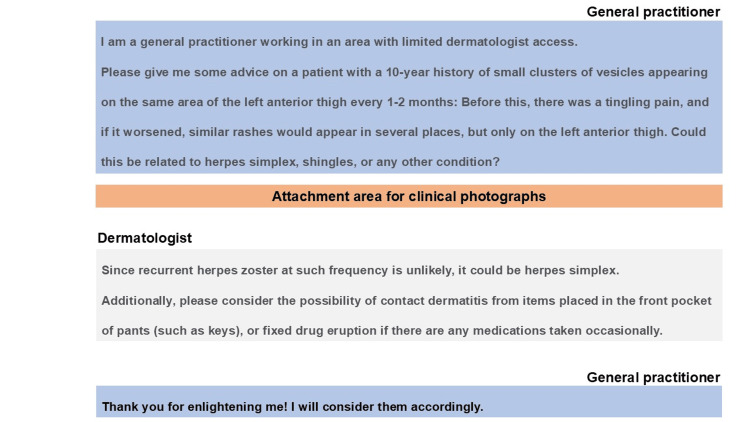
The conceptual visual design of our internet/application-based e-consult system

The number of cases where a plan was determined using e-consults was 83, accounting for 91% of the total 91 cases. All these cases solely established a patient management plan through e-consults, with GPs consistently following hospital specialists' advice. The number of cases for which a plan was determined through e-consults, along with the respective rates for each hospital specialist, were as follows: 52 cases (90%) by internal medicine subspecialists, 16 cases (89%) by minor specialists, 8 cases (100%) by general internal medicine specialists, and 7 cases (100%) by surgery. Patients’ problems related to internal medicine were most commonly addressed through e-consults; the patient plans were determined via e-consults in more than 90% of cases (Table 2 and Figure 3).

The level of GP satisfaction with the e-consult process was high, with a median satisfaction score of 5 on a 5-point Likert scale. Most e-consults received their initial response within two hours. The number of chats required for hospital specialists to respond was very low, with a median of only one chat being necessary (Table 2).

The detail of the divisions of the hospital specialists

Among the internal medicine subspecialties, rheumatology was the most common, with 15 cases (16%), followed by infectious diseases with 10 cases (11%), pulmonology with 7 cases (8%), neurology with 6 cases (7%), and endocrinology with 6 cases (7%). Among the minor specialists, dermatology was the most common, with eight cases (9%), followed by pediatrics with three cases (3%) (Table 3).

Patients’ problems that can be dealt with via e-consults

A wide range of patients’ problems were addressed via e-consults, and these problems varied among hospital specialists (Table 4). For internal medicine specialists, fever of unknown origin was the most common reason for consultation, followed by interstitial pneumonia, diabetes mellitus, and COVID-19 vaccination.

Regarding minor specialists, dermatology consultations for rashes were the most common. For general internal medicine physicians, all e-consults were for undiagnosed symptoms rather than diagnosed conditions, and the diagnostic plan for each patient was determined solely through the e-consults (Table 4).

## Discussion

Our study found that 52% of cases had their management plans determined via the Internet/application-based e-consult system alone, and in 91% of cases, future diagnostic and treatment plans were determined by e-consults. The Internet/application-based e-consult system may have the potential to enable GPs to consult various hospital specialists to address a wide range of medical issues. However, our study is a descriptive study and, therefore, could not evaluate the effectiveness of the internet/application-based e-consult system.

GPs have shown a high-resolution rate when using the Internet/application-based e-consult system. Previous studies showed that implementing e-consults in GP clinics allowed specialists to respond without additional information in 89% of cases, and over 90% of cases received a hospital specialist response within 15 minutes [18]. Another previous study found that the rate of GPs adhering to hospital specialists' recommendations via e-consults was 82% [19]. The short response time and high adherence rate in this study were consistent with these previous studies. A unique feature of the Internet/application-based e-consult system is its chat-based format. It enables GPs to engage in real-time, interactive discussions with hospital specialists, allowing them to refine their questions. The chat-based interaction may help GPs gain a deeper understanding, potentially making it easier to resolve patient problems.

Using this Internet/application-based e-consult system, GPs can potentially resolve a wide range of patients’ problems easily and effectively. In our study, GPs used e-consults in various clinical areas, with particularly high rates of use in infectious diseases, rheumatology, dermatology, and diagnostically challenging cases. In Japan, hospital-based GPs often provide COVID-19 care in hospitals in the absence of infectious disease specialists [20]. In our study, GPs used e-consults for COVID-19 treatment and vaccination, demonstrating their potential usefulness in the management of COVID-19 care [21]. In addition, the usefulness of e-consults has been reported in dermatology and rheumatology, being consistent with our study results [22,23]. Previous studies reported that using e-consults by GPs reduces unnecessary face-to-face referrals and promotes efficient and timely care [24,25]. Our e-consult system may have the potential to reduce unnecessary remote consultations and potentially improve the quality of care provided by GPs.

Internet/application-based e-consult systems are one of the digital health tools that have the potential to improve the quality of care provided by GPs. The Clinical Decision Support System (CDSS) is a well-known digital health tool for improving the clinical practice of GPs [26]. Previous studies in Japan have shown that the use of CDSSs by GPs can increase bisphosphonate prescription rates for steroid-induced osteoporosis and help monitor potentially harmful medications [27-28]. However, using CDSS carries the risk of alert fatigue, whereby frequent or unspecific alerts may cause clinicians to overlook or disregard clinically important information [29]. In addition, while CDSS can support adherence to guideline-based care, the system is difficult to update, and there are significant costs associated with implementation [25]. In contrast, an Internet/application-based e-consult system provides easy access to up-to-date knowledge and can be implemented at a low cost. While generative artificial intelligence (AI) can effectively solve simpler medical problems in primary care, its effectiveness in complex medical problems remains limited [30]. An Internet/application-based e-consult system may have the potential to help GPs solve complex medical problems. However, internet/application-based e-consult systems, unlike CDSS or generative AI systems, are limited in their ability to address medical problems that have not already been identified by GPs.

Our study had some limitations. First, this study was conducted only in Japan, which may limit the generalizability of our findings to other countries with different healthcare systems. In addition, the system may be difficult to implement in regions where Internet resources are scarce. However, because the Internet/application-based e-consult system can be operated using only a standard Web browser and does not require expensive or specialized technology, it may still be feasible in a variety of settings. Second, problem resolution was evaluated based on analysis of web chat information and GP reports; we did not employ any follow-up to verify a patient's outcome after the e-consult. However, GPs using the e-consult system considered the patients’ problems to have been resolved and accepted the e-consult recommendations in most cases. Future research will need to assess the actual acceptance rate at which GPs implement e-consult recommendations in their clinical practice. Third, the sample size of our study was small; therefore, we could not thoroughly account for potential biases, missing data, or unmeasured confounding variables. Further studies with sufficiently large sample sizes are needed to address these issues. Fourth, we did not compare an Internet/application-based e-consult system with a traditional e-consult system. Hence, the effects of the Internet/application-based e-consult system remain unclear. Fifth, although the study was conducted across Japan, the sample size was small, limiting its external validity. Sixth, our study focused on e-consult cases initiated by GPs, making it impossible to compare with e-consult cases where the users were not GPs. In Japan, e-consults are often used by non-GPs. Seventh, this is a descriptive study and was not designed to assess the association between exposure and outcome. Therefore, p-values and confidence intervals were not calculated. Future research should be designed to address these limitations. Eighth, this study was conducted during the COVID-19 pandemic, and thus, we cannot entirely exclude its potential influence. However, only four of the consultations addressed COVID-19-related issues, three of which pertained to vaccinations. Therefore, we believe that the pandemic had a limited impact on our study. Ninth, because the Internet/application-based e-consult system was not integrated with electronic health records, it could not be implemented at the facility level. As a result, improvements in patient outcomes or costs before and after implementation could not be evaluated.

Therefore, future research using an Internet/application-based e-consult system with a larger sample size and a robust study design capable of assessing the association between exposure and outcome while controlling for potential confounders is needed.

## Conclusions

In conclusion, although the scope of participants and settings in this study was limited, our findings suggest that an Internet/application-based e-consult system can help GPs address a wide range of medical issues, with 52% of cases resolved solely through e-consult and 91% having their diagnostic and treatment plans determined entirely through e-consult. While these results serve as an important benchmark for future research on e-consults in Japan, further studies with large samples are needed to confirm the broader applicability and benefits of this system.

## Appendices

**Table 3:** Divisions of hospital specialists

Specialist divisions in hospitals	E-consult cases in the study (N=91)
Internal medicine subspecialties, n	58
Rheumatology, n (%)	15 (16)
Infectious diseases, n (%)	10 (11)
Pulmonology, n (%)	7 (8)
Neurology, n (%)	6 (7)
Endocrinology and metabolism, n (%)	6 (7)
Hematology, n (%)	4 (4)
Nephrology, n (%)	3 (3)
Gastroenterology, n (%)	2 (2)
Cardiovascular, n (%)	2 (2)
Allergology, n (%)	2 (2)
Geriatric, n (%)	1 (1)
Minor specialists, n	18
Dermatology, n (%)	8 (9)
Pediatrics, n (%)	3 (3)
Emergency care, n (%)	2 (2)
Urology, n (%)	2 (2)
Ophthalmology, n (%)	1 (1)
Otorhinolaryngology, n (%)	1 (1)
Palliative care, n (%)	1 (1)
General internal medicine, n (%)	8 (9)
Surgery, n	7
Orthopedic surgery, n (%)	2 (2)
Gastroenterological surgery, n (%)	2 (2)
Neurosurgery, n (%)	1 (1)
Pediatric surgery, n (%)	1 (1)
Cardiovascular surgery, n (%)	1 (1)

**Table 4:** Number of patients’ problems and classification by hospital specialists

Patients’ problems	Number of problems (N=91)
Internal medicine subspecialties, n	58
Fever of unknown origin, n (%)	10 (11)
Interstitial pneumonia, n (%)	5 (5.5)
COVID-19 vaccination, n (%)	3 (3)
Diabetes mellitus, n (%)	3 (3)
Antibiotic-resistant bacteria, n (%)	2 (2)
Liver dysfunction, n (%)	2 (2)
Rheumatoid arthritis, n (%)	2 (2)
Aphasia, n (%)	1 (1)
Arthritis, n (%)	1 (1)
Aspiration, n (%)	1 (1)
Atrial fibrillation, n (%)	1 (1)
Cerebral infarction, n (%)	1 (1)
Chronic cough, n (%)	1 (1)
Chronic renal failure, n (%)	1 (1)
COVID-19, n (%)	1 (1)
Disorder of consciousness, n (%)	1 (1)
Electrocardiogram abnormalities, n (%)	1 (1)
Hypocalcemia, n (%)	1 (1)
Iron-deficiency anemia, n (%)	1 (1)
Lymphocytosis, n (%)	1 (1)
Meningitis, n (%)	1 (1)
Motor dysfunction, n (%)	1 (1)
Multiple abscesses, n (%)	1 (1)
Nephrotic syndrome, n (%)	1 (1)
Parkinson's disease, n (%)	1 (1)
Pleural effusion, n (%)	1 (1)
Pneumonia, n (%)	1 (1)
Polycystic kidney disease, n (%)	1 (1)
Positive syphilis test, n (%)	1 (1)
Positive urinalysis urobilinogen, n (%)	1 (1)
Post-streptococcal reactive arthritis, n (%)	1 (1)
RS3PE syndrome, n (%)	1 (1)
Scleroderma, n (%)	1 (1)
Secondary hypertension, n (%)	1 (1)
Upper limb pain, n (%)	1 (1)
Vasculitis, n (%)	1 (1)
Vitamin B12 deficiency, n (%)	1 (1)
Graves' disease, n (%)	1 (1)
Minor specialists, n	18
Rash, n (%)	5 (5.5)
Animal bite, n (%)	2 (2)
Ingrown toenail, n (%)	1 (1)
Conjunctivitis, n (%)	1 (1)
Microscopic hematuria, n (%)	1 (1)
Post-ejaculatory pain, n (%)	1 (1)
Prostate cancer, n (%)	1 (1)
Colorectal cancer, n (%)	1 (1)
Onychomycosis, n (%)	1 (1)
Hypocalcemia, n (%)	1 (1)
Pruritus, n (%)	1 (1)
Perinasal infection, n (%)	1 (1)
Vomiting, n (%)	1 (1)
General internal medicine, n	8
Dizziness, n (%)	2 (2)
Numbness, n (%)	1 (1)
Motor dysfunction, n (%)	1 (1)
Oculomotor dysfunction, n (%)	1 (1)
Fatigue, n (%)	1 (1)
Rash, n (%)	1 (1)
Hoarseness, n (%)	1 (1)
Surgery, n	7
Subdural hematoma, n (%)	1 (1)
Short bowel syndrome, n (%)	1 (1)
Enteritis, n (%)	1 (1)
Rash, n (%)	1 (1)
Abdominal aortic aneurysm, n (%)	1 (1)
Radial fracture, n (%)	1 (1)
Numbness, n (%)	1 (1)
